# RETRACTION: A Novel Oxidovanadium (IV)‐Orotate Complex as an Alternative Antidiabetic Agent: Synthesis, Characterization, and Biological Assessments

**DOI:** 10.1155/bmri/9768273

**Published:** 2026-06-10

**Authors:** BioMed Research International

**RETRACTION**: A. M. Naglah, M. A. Al‐Omar, A. A. Almehizia, M. A. Bhat, W. M. Afifi, A. S. Al‐Wasidi, J. Y. Al‐Humaidi, M. S. Refat, “A Novel Oxidovanadium (IV)‐Orotate Complex as an Alternative Antidiabetic Agent: Synthesis, Characterization, and Biological Assessments,” *BioMed Research International*, 2018, 8108713, 10.1155/2018/8108713

The above article, published online on 23 December 2018 in Wiley Online Library (http://wileyonlinelibrary.com), has been retracted by agreement between the journal Editor‐in‐Chief, Yoel Klug; and John Wiley & Sons Ltd. The retraction has been agreed due to concerns identified in Figure 6, initially reported on PubPeer [1]. Specifically:

• In Figure 6, panel B appears highly similar to the top right (BDL + EX527) panel in Figure 1a of [2]. This is apparent after adjusting the contrast and rotating the image 180 degrees.
• In Figure 6, panel C appears highly similar to the bottom left (BDL + sil20 + EX527) panel in Figure 1a of [2]. This is apparent after adjusting the contrast and rotating the image 180 degrees.
• In Figure 6, panel C appears highly similar to the top middle (BDL) panel in Figure 1a of [2]. This is apparent after adjusting the contrast and rotating the image 180 degrees

After assessment of the concerns and the author’s response, the editorial board have confimed that the results and conclusions can no longer be considered reliable, and the article is retracted.

The authors disagree with the retraction.
